# The outcomes of transcatheter adrenal ablation in patients with primary aldosteronism: a systematic review and meta-analysis

**DOI:** 10.1186/s12902-023-01356-9

**Published:** 2023-05-08

**Authors:** Shunfan Yang, Guoliang Wang, Nanfang Li, Qing Zhu

**Affiliations:** Hypertension Center of People’s Hospital of Xinjiang Uygur Autonomous Region, Xinjiang Hypertension Institute, NHC Key Laboratory of Hypertension Clinical Research, Key Laboratory of Xinjiang Uygur Autonomous Region “Hypertension Research Laboratory”, Xinjiang Clinical Medical Research Center for Hypertension (Cardio-Cerebrovascular) Diseases, People’s Hospital of Xinjiang Uygur Autonomous Region, Urumqi, 830000 Xinjiang Uygur Autonomous Region China

**Keywords:** Primary aldosteronism, Transcatheter adrenal ablation, Clinical success, Meta-analysis

## Abstract

**Background:**

The use of transcatheter adrenal ablation as an alternative treatment for primary aldosteronism (PA) patients remains a subject of debate, with outcomes varying widely across existing studies. This meta-analysis aims to evaluate the results of adrenal ablation and estimate the effectiveness and safety of this therapeutic approach.

**Methods:**

A comprehensive search was conducted across PubMed, Embase, and Cochrane Library databases for studies published up to October 2022. Outcomes analyzed included the combined clinical success rate, biochemical success rate, and complication rate, which were assessed using a random-effects model.

**Results:**

Five studies, comprising 234 PA patients, were included in the analysis. The combined clinical success rate was 74% (95% CI: 69%-79%), and the biochemical success rate was 74% (95% CI: 53%-95%). Subgroup analysis revealed that the combined clinical success rate from Unilateral PA (72%, 95% CI: 46%-98%) was similar to the rate from Unilateral + Bilateral (73%, 95% CI: 52.0%-94.0%), while the clinical success rate of the PASO subgroup (78%, 95% CI: 66.0%-89.0%) was higher than the rate of other criteria (51%, 95% CI: 40.0%-63.0%). The combined complication rates were as follows: mild fever, 23% (95% CI: 12%-33%); back pain, 84% (95% CI: 77%-91%); and pleural effusion, 9% (95% CI: 0%-18%). All complications resolved within one week following the procedure. No late complications or ablation-related deaths were reported.

**Conclusions:**

Transcatheter adrenal ablation for PA patients is safe and demonstrates a relatively high clinical success rate. Presently, this approach is suitable for PA patients who are unwilling to undergo surgery or receive long-term mineralocorticoid receptor antagonist (MRA) treatment.

**Systematic Review registration:**

INPLASY, identifier 2022110076

**Supplementary Information:**

The online version contains supplementary material available at 10.1186/s12902-023-01356-9.

## Introduction

Primary aldosteronism (PA) is a prevalent secondary hypertension, affecting over 10% of patients with general hypertension and up to 20% of those with resistant hypertension [1–3]. Patients with PA face higher risks of severe cardiovascular and renal complications compared to those with essential hypertension [4–6]. The most common PA subtypes are aldosterone-producing adenoma (APA), unilateral adrenal hyperplasia (UAH), and idiopathic hyperaldosteronism (IHA). For patients with APA and UAH, unilateral adrenalectomy is recommended, as it can cure 30 to 60 percent of hypertension cases [7]. In contrast, adrenalectomy rarely rectifies hypertension in bilateral IHA, making medication the preferred treatment [1]. However, some unilateral PA patients refuse adrenalectomy due to surgical risks, while others may not be suitable candidates for surgery due to severe illnesses. Although medication is advised for IHA patients, some may experience adverse reactions to mineralocorticoid receptor antagonists (MRAs). Consequently, an alternative treatment is needed in these cases.

Transcatheter adrenal ablation is an interventional procedure involving selective ethanol injection into the adrenal artery to ablate part of the adrenal tissue. Past studies have demonstrated promising outcomes for this technique in treating adrenal tumors and hemorrhages [8, 9]. Recently, transcatheter adrenal ablation has emerged as a potential alternative treatment for patients with unilateral PA and IHA [10, 11].

Despite its perceived effectiveness in treating PA, most evidence supporting transcatheter adrenal ablation comes from small sample size studies or case reports, with results varying widely. Furthermore, the use of transcatheter adrenal ablation as an alternative treatment for PA patients remains controversial. By collecting and analyzing the outcomes of existing studies on transcatheter adrenal ablation, this meta-analysis aims to evaluate the effectiveness and safety of this therapeutic method. Ultimately, this analysis may significantly contribute to understanding alternative treatments for PA, potentially impacting clinical practice and patient outcomes.

## Methods

### Literature search strategy

This systematic review and meta-analysis followed the Preferred Reporting Items for Systematic Reviews and Meta-analyses (PRISMA) Guidelines [12]. We searched papers in Pubmed, Embase and Cochrane library databases published up to October 2022 comprehensively. We used the following keywords to recognize possibly relevant studies from all databases: “Hyperaldosteronism OR Aldosteronism OR Primary Hyperaldosteronism OR Hyperaldosteronism, Primary” AND “Ablation Technique OR Embolization, Therapeutic OR Ablation OR Technique, Ablation OR Embolization OR Embolotherapy OR Therapeutic, Embolization” (Supplementary Table 1).

### Inclusion and exclusion criteria

We used the following criteria for screening of the papers:1) Standard diagnostic and confirmatory test was employed to confirm PA [1]. 2) The patients of studies must perform adrenal venous blood sampling before transcatheter adrenal ablation. 3) The study should provide the clinical success rate, or enough data for inference of the outcome. 4) The definition of outcome should be clearly specified in the papers and the criteria of ablation success include cure and significant remission. 5) Study included should provide a clear follow-up period. 6) The study design include RCT and non-randomized studies. Exclusion criteria were review articles, case reports, editorial comments, conference abstracts and letters. For multiple articles from the same set of data, the article with the largest amount of data is considered.

### Extraction of data

All data were extracted separately by the two independent reviewers. The following data were extracted for each study: (1) general manuscript information: first author, year of publication, country; (2) patients characteristics: sample size, age, gender; (3) Outcomes of effectiveness: clinical success rate, biochemical success rate, complication(included fever, back pain and pleural effusion); (4) major complications. In the event of a dispute between the reviewers, a discussion was conducted in order to arrive at consensus.

### Assessment of bias risk

The Methodological index for non-randomized studies (MINORS) tool was used to assess risk of bias [13]. The ideal score being 16 for non-comparative studies and 24 for comparative studies. The bias risk was appraised by two independent reviewers, and discrepancies were resolved in consensus between the two reviewers.

### Statistical analysis

All statistical calculations were performed using STATA, version 17.0 (STATA, College Station, TX). Rate of outcomes, with their 95% confidence interval (95%CI), were used as the effect size. Given the high heterogeneity between studies, we used the DerSimonian and Laird method in generating the random effects models for the estimation of pooled rate. Statistical heterogeneity among studies was calculated using the *I* ^2^ statistic, *I* ^2^ > 50% is regarded as high-level heterogeneity [14]. We performed subgroup analyses to explore the potential sources of heterogeneity, including PA subtype(only unilateral and unilateral + bilateral) and outcome criteria(PASO and other). Sensitivity analyses of success rates were conducted. Publication bias could not be evaluated in ten or fewer studies as they lacked test power [15]. *P* values of less than 0.05 were regarded as statistically significant.

## Results

### Literature search and bias assessment

**Fig. 1:**
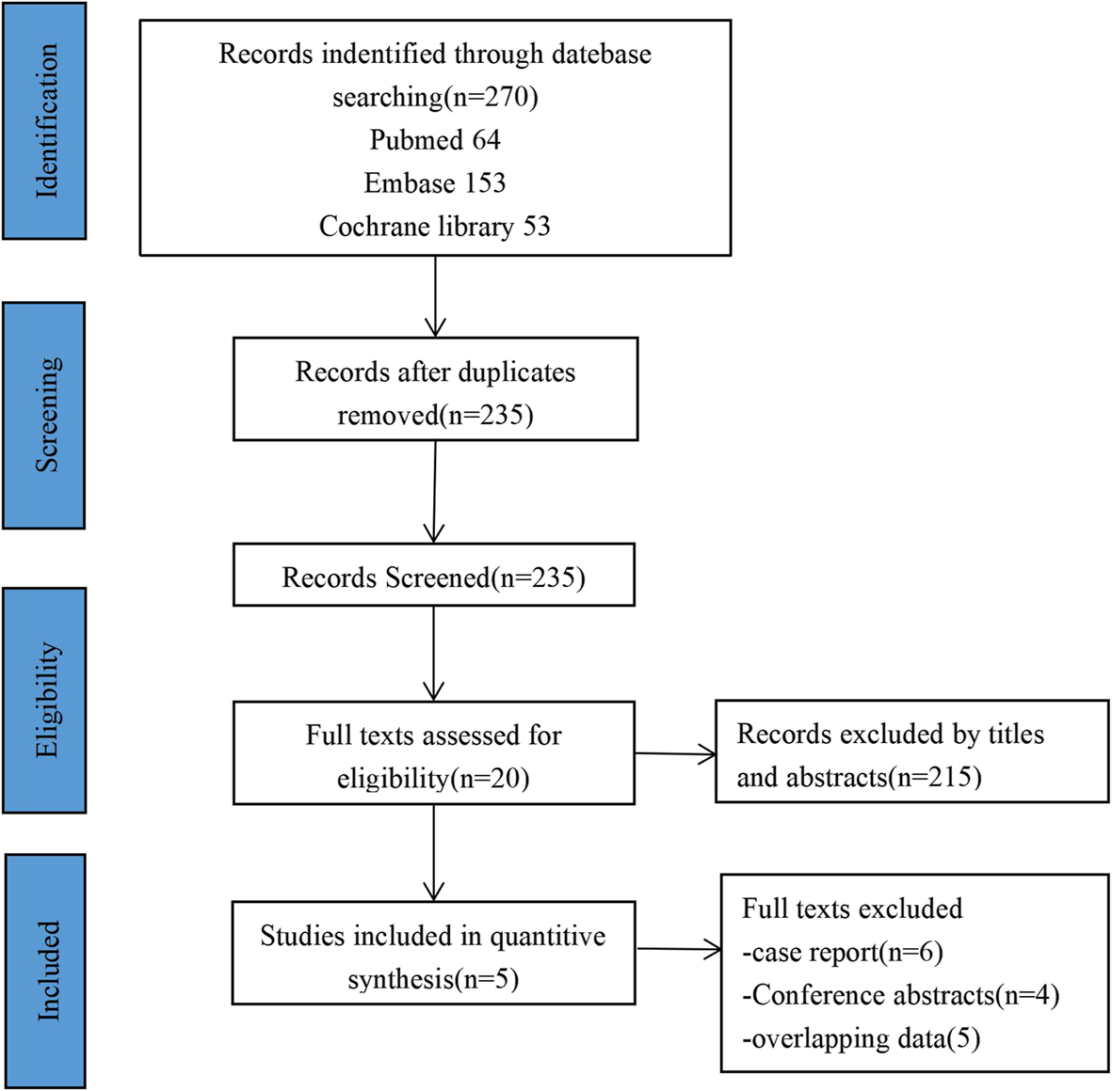
Flowchart of study selection

The literature search process is illustrated in Fig. 1. A total of 270 articles were retrieved from database searches, including 64 from PubMed, 153 from Embase, and 53 from the Cochrane Library. From these articles, 35 duplicates were removed. The titles and abstracts of the remaining 235 articles were screened, and an additional 215 articles that were not relevant to the study's objectives were excluded. The full texts of the remaining 20 articles were reviewed. These articles were excluded for the following reasons: case report (*n* = 6), conference abstracts (*n* = 4), and overlapping data (*n* = 5). Ultimately, five studies comprising 234 PA patients were included in this meta-analysis [10, 11, 16–18] (Fig. 1). All five studies were non-randomized cohort studies. The risk of bias was assessed using the MINORS tool (Table 1). The mean MINORS score for comparative studies was 19.5 ± 0.7 out of 24, while the MINORS score for non-comparative studies was 10.7 ± 0.6 out of 16.

**Table 1:** The risk of bias assessment of non-randomized studies (MINORS)

Methodological items for non-randomized studies	Dong 2021 [10]	Hokotate 2003 [16]	Sun 2022 [17]	Zhang 2020 [11]	Zhou 2022 [18]
1.A clearly stated aim	2	2	2	2	2
2.Inclusion of consecutive patients	1	1	2	2	2
3.Prospective collection of data	2	2	2	2	2
4.Endpoints appropriate to the aim of the study	2	2	2	2	2
5.Unbiased assessment of the study endpoint	0	0	0	0	0
6.Follow-up period appropriate to the aim of the study	2	2	2	2	2
7.Loss to follow up less than 5%	2	2	2	0	2
8.Prospective calculation of the study size	0	0	0	0	0
9.An adequate control group	0	0	1	0	2
10.Contemporary groups	0	0	2	0	2
11.Baseline equivalence of groups	0	0	2	0	2
12.Adequate statistical analyses	0	0	2	0	2
Total score	11	11	19	10	20

### Study characteristics

The main characteristics of the included studies are presented in Table 2. All studies were conducted in Asian populations. Each study provided data on age, sex, and duration of follow-up. Three studies [11, 17, 18] used the Primary Aldosteronism Surgical Outcome (PASO) [19] as the criterion for clinical success and biochemical success, which included complete and partial success. The remaining two studies [10, 16] employed other criteria and lacked data on biochemical success. In the study by Dong, et al. [10], only marked improvement was considered as clinical success because moderate improvement was regarded as no change according to other criteria.

**Table 2:** Characteristics of the included studies

Study, Years	Country	Study type	Ablation patients	Mean age ± SD (year)	Male (%)	PA subtype	Outcome criteria	follow-up times(months)	Clinical success rate (%)	Biochemical success rate (%)
Dong 2021 [10]	China	Prospective	39	41.0 ± 9.4	29 (70.7)	bilateral	Other^a^	≥ 12	0.46	NA
Hokotate 2003 [16]	Japan	Prospective	33	47.0 ± 8.0	25 (75.8)	Unilateral	Other^b^	≥ 6	0.58	NA
Sun 2022 [17]	China	Prospective	52	45.0 ± 11.9	23 (44.2)	Unilateral	PASO	≥ 6	0.85	0.90
Zhang 2020 [11]	China	Prospective	36	48.4 ± 12.5	21 (58.3)	Unilateral + bilateral	PASO	≥ 6	0.61	0.44
Zhou 2022 [18]	China	Prospective	74	54.2 ± 10.9	19 (25.7)	Unilateral + bilateral	PASO	≥ 6	0.82	0.82

*NA* Not available

PASO: Complete clinical success is defined as normal blood pressure without the aid of antihypertensive medication. Partial clinical success is defined as the same blood pressure as before ablation with less antihypertensive medication or a reduction in blood pressure with either the same amount or less antihypertensive medication. Absent clinical success is defined as unchanged or increased blood pressure with either the same amount or an increase in antihypertensive medication. Complete biochemical success is defined as correction of hypokalaemia (if present pre-ablation) and normalisation of the aldosterone-to-renin ratio; in patients with a raised aldosterone-to-renin ratio post surgery, aldosterone secretion should be suppressed in a confirmatory Test. Partial biochemical success is defined as correction of hyqpokalaemia (if present pre-surgery) and a raised aldosterone-to-renin ratio with one or both of the following (compared with pre-surgery): decrease in baseline plasma aldosterone concentration ≥ 50%; or abnormal but improved post-surgery confirmatory test result. Absent biochemical success is defined as persistent hypokalaemia (if present pre-surgery) or persistent raised aldosterone-to-renin ratio, or both, with failure to suppress aldosterone secretion with a post-surgery confirmatory test

Other^a^: Marked improvement is defined as a reduction in the home systolic or diastolic blood pressure by > 20 mmHg or > 10 mmHg, respectively, without the use of MRA, while the dosage of other antihypertensive drugs remained unchanged or reduced. Moderate improvement is defined as a reduction in home systolic or diastolic blood pressure by 10–20 mmHg or 5–10 mmHg, respectively, without the use of MRA or with its dose reduced to half, while the dosage of other antihypertensive drugs remained unchanged or reduced

Other^b^: Normalization is defined as blood pressure lower than 140/90 mm Hg without the use of antihypertensive medication. Marked improvement is defined as a decrease of at least 20 mmHg in thesystolic or diastolic blood pressure either without the use of antihypertensive medication or with a decrease in antihypertensive medication dosage

### Clinical success and bichemical success rate of adrenal ablation

**Fig. 2:**
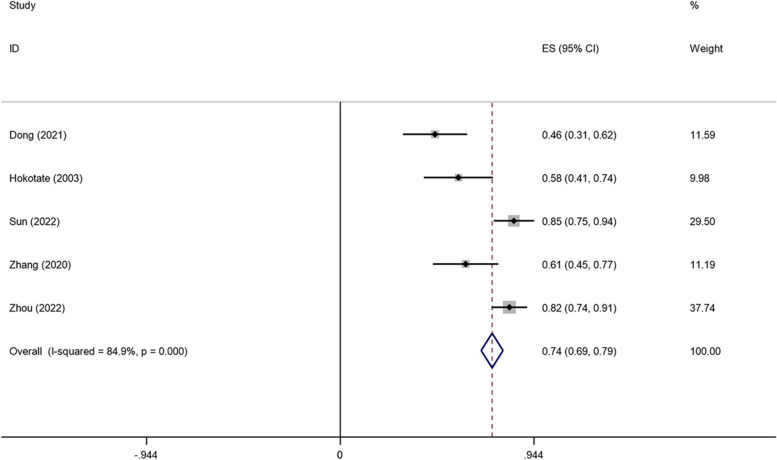
Forest plots of clinical success of adrenal ablation

**Fig. 3:**
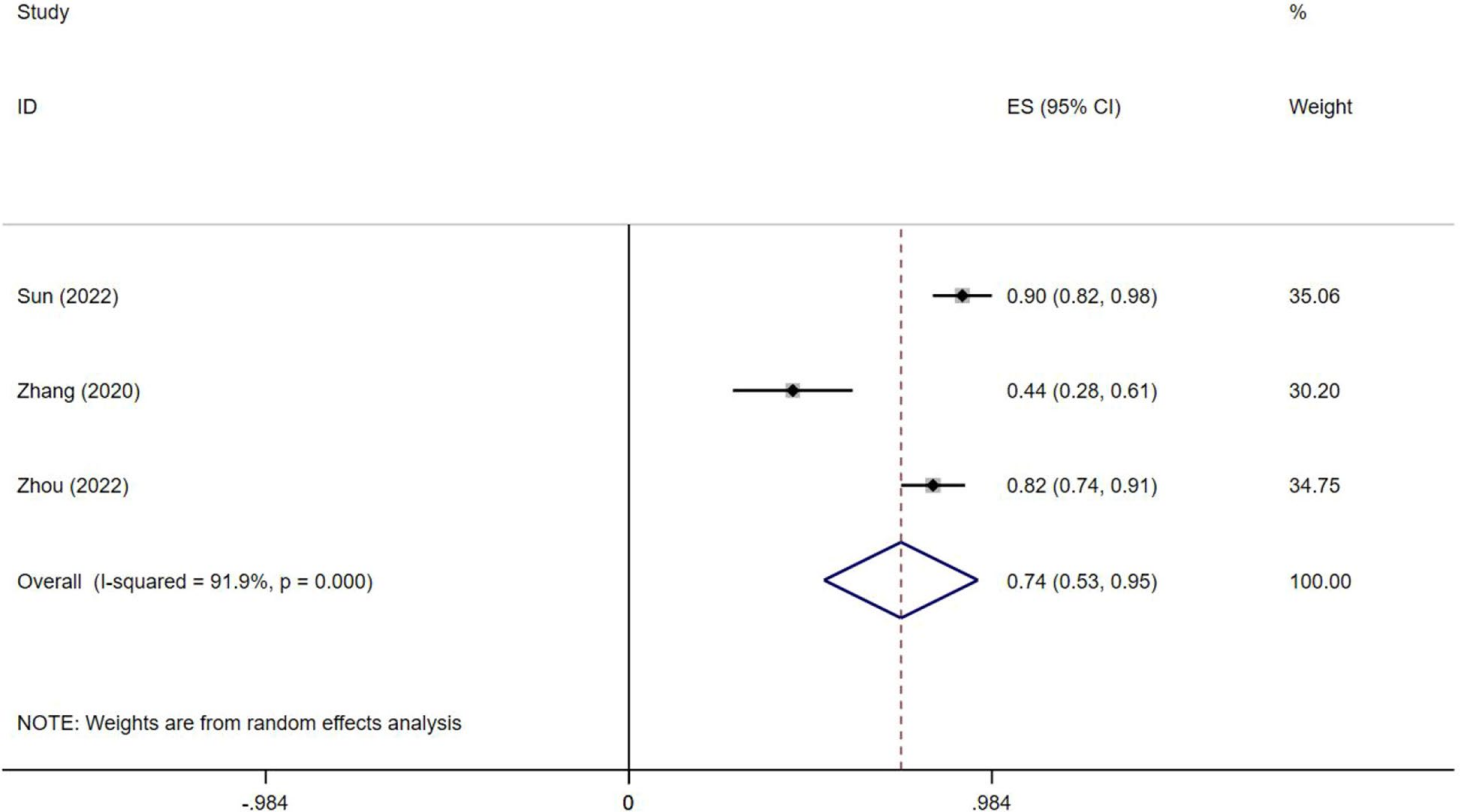
Forest plots of biochemical success rate of adrenal ablation

The clinical success rate of transcatheter adrenal ablation in PA ranged from 58 to 85% and the combined clinical success rate was 74% (95% CI: 69%-79%; Fig. 2). The biochemical success rate ranged from 44 to 90% and the combined biochemical success rate was 74% (95%CI: 53%-95%; Fig. 3). Both the clinical success rate (I^*2*^ = 84.9%, *P* = 0.000) and biochemical success rate (I^*2*^ = 91.9%, *P* = 0.000) indicated a high level of heterogeneity between the studies.

PA patients can be further stratified according to PA subtype and outcome criteria. The combined clinical success rate of the subgroups is presented in Table 3. The combined clinical success rate for Unilateral PA (72%, 95% CI: 46%-98%) was similar to the rate for Unilateral + Bilateral (73%, 95% CI: 52.0%-94.0%), (*P* = 0.831). However, the clinical success rate for the PASO subgroup (78%, 95% CI: 66.0%-89.0%) was higher than the rate for other criteria (51%, 95% CI: 40.0%-63.0%), (*P* < 0.05).

**Table 3:** Clicinal success rate of adrenal ablation by different categories

Category	Subgroup	NO. of Studies	Ablation patients	Clicinal success rate (95% CI)	I^2^ (%)	*P*-value of Q-test	*P*-value of group differences
Subtype of PA	Unilateral	2	85	0.72(0.46,0.98)	86.5	0.007	0.831
	Unilateral + bilateral	2	110	0.73(0.52,0.94)	81.2	0.021	
Outcome criteria	PASO	3	162	0.78(0.66,0.89)	69.5	0.038	0.000
	Other criteria	2	72	0.51(0.40,0.63)	0.0	0.330	

Sensitivity analyses were conducted for the clinical success rate and biochemical success rate. When any one study was excluded, the results remained consistent (Supplementary Fig. 1, Supplementary Fig. 2).

### Complication

Complications during follow-up were detailed in all five included studies, primarily consisting of back pain, mild fever, pleural effusion, and gastrointestinal symptoms. Major complications were quantified in 107 PA patients across two studies [16, 18]. The combined occurrence rates were as follows: mild fever at 23% (95% CI: 12%-33%), back pain at 84% (95% CI: 77%-91%), and pleural effusion at 9% (95% CI: 0%-18%) (Table 4). All complications resolved within one week following the procedure. No late complications or ablation-related deaths were reported.

**Table 4:** Occurrence rate of main complication of adrenal ablation

Complication	NO. of Studies	Ablation patients	Occurrence rate	I^2^ (%)	*P*-value of Q-test
Fever	2	107	0.23(0.12,0.33)	34.6	0.216
Flank or back pain	2	107	0.84(0.77,0.91)	0.0	0.074
pleural effusion	2	107	0.09(0.00,0.18)	51.7	0.150

## Discussion

Currently, studies have demonstrated that transcatheter adrenal ablation can serve as a vital alternative treatment for PA patients who refuse surgery or medical treatment. However, the reported success rates of adrenal ablation exhibit significant disparities. Some experts also express concerns regarding the efficacy and safety of adrenal ablation for PA patients. Consequently, a systematic review of published studies on the success rate of transcatheter adrenal ablation for PA was conducted.

Unilateral PA patients can be cured or significantly relieved after unilateral laparoscopic adrenalectomy [7, 19]. An international cohort of adrenalectomy for unilateral PA indicated complete and partial clinical success rates of 84%, with a biochemical success rate of 94% [19]. Five studies of adrenal ablation were included, encompassing 234 PA patients. The pooled clinical success rate was 74% (95% CI: 69%-79%), and the combined biochemical success rate was 74% (95% CI: 53%-95%). The clinical success rate of adrenal ablation is comparable to that of adrenalectomy, though the biochemical success rate is lower, which is unsurprising since ablation only partially destroys adrenal function.

The success rate of unilateral or bilateral adrenalectomy in bilateral PA patients is low [20]. Medical therapy is recommended for these patients. However, some patients are intolerant to MRA-related adverse reactions. Partially resistant hypertension patients refuse to take excessive antihypertensive drugs and prefer to alleviate their condition through adrenal ablation. Subgroup analysis revealed no significant difference in the clinical success rate of adrenal ablation for the unilateral + bilateral group compared to the unilateral group. Although the remission rate of Dong 2021, which only included bilateral PA patients, was lower than in other studies, the plasma aldosterone level was significantly reduced after ablation [10]. A meaningful reduction of aldosterone levels may decrease the risk of cardiovascular disease, even if the blood pressure reduction is not significant [21, 22]. This finding suggests that transcatheter adrenal ablation can be employed not only in unilateral PA but also in bilateral PA, where prevalence is higher.

Studies indicate that adrenal ablation is effective for PA patients, but the heterogeneity of success rates is high between studies. The Primary Aldosteronism Surgical Outcome (PASO) [19] is a consensus criterion for outcomes and follow-up of adrenalectomy for unilateral primary aldosteronism. Among the included studies, three used PASO criteria for outcomes, while two employed other criteria. Heterogeneity of clinical success rate was reduced after performing subgroup analysis by different outcome criteria. Differences in outcome criteria may contribute to the observed heterogeneity. The clinical success rate of the PASO group was found to be higher than that of the other criteria group. However, in the unilateral and unilateral + bilateral subgroup, outcome heterogeneity remains high.

Studies have demonstrated that female and younger PA patients are more likely to achieve clinical success following unilateral adrenalectomy [19]. As a result, differences in the baseline data of patients included between studies may contribute to the high heterogeneity of outcomes observed after adrenal ablation. Additionally, the adrenal gland is supplied by three arteries, each of which serves different areas of the gland. Adrenal ablation demands high technical expertise from interventional physicians, and there is currently no method to evaluate the extent of ablation during the process. At present, there is no uniform standard for the amount of alcohol used during ablation, and differences in the quantity used may result in varying outcomes. For APA patients, adenomas are likely to receive blood supply from two or more vessels, so embolizing only one vessel is the main cause of disease recurrence [16]. These factors may be other sources of high heterogeneity.

Complications associated with adrenal ablation resolved within one week, with no serious long-term complications reported. Notably, no adrenal cortical dysfunction, a common complication in adrenalectomy, was observed after adrenal ablation [23, 24].

There are several limitations to this study: the included studies were non-RCTs. The studies lacked a uniform control group (drug therapy or adrenalectomy group), and only the combined rate of outcomes was analyzed. Most of the studies had small sample sizes. Some studies did not use PASO criteria to assess outcomes, resulting in high heterogeneity. Lastly, publication bias was not assessed due to the low number of studies included. In the future, more randomized controlled studies with larger samples are needed to demonstrate the effectiveness and safety of adrenal ablation.

## Conclusion

This meta-analysis indicates that adrenal ablation for PA is safe and effective. Currently, this approach is suitable for PA patients who are unwilling to accept surgery and long-term MRA treatment. The high heterogeneity between studies necessitates higher-level evidence and uniform efficacy evaluation standards.

## Supplementary Information

**Additional file 1: Table S1.**

**Additional file 2: Figure S1.**

**Additional file 3: Figure S2.**

## Data Availability

The datasets used and analysed during the current study available from the corresponding author on reasonable request.

## Abbreviations

PA: Primary aldosteronism
APA: Aldosterone-producing adenoma
UAH: Unilateral adrenal hyperplasia
IHA: Idiopathic hyperaldosteronism
MINORS: Methodological index for non-randomized studies
PASO: Primary Aldosteronism Surgical Outcome
