# Supplementary material for: The outcomes of transcatheter adrenal ablation in patients with primary aldosteronism: a systematic review and meta-analysis
Source: BMC Endocr Disord. 2023 May 8;23:103. doi: 10.1186/s12902-023-01356-9 (PMC10165838; doi:10.1186/s12902-023-01356-9)
Supplement: Supplementary file 1 — Additional file 1: Table S1. [file 12902_2023_1356_MOESM1_ESM.docx]

PubMed search strategy

| Search number | Search Details | Results |
| --- | --- | --- |
| 3 | ("Hyperaldosteronism"[MeSH Terms] OR "Aldosteronism"[Title/Abstract] OR "primary hyperaldosteronism"[Title/Abstract] OR "hyperaldosteronism primary"[Title/Abstract]) AND ("ablation technique*"[MeSH Terms] OR ("embolization*"[All Fields] AND "therapeutics"[MeSH Terms]) OR "Ablation"[Title/Abstract] OR ("technique*"[All Fields] AND "Ablation"[Title/Abstract]) OR "Embolization"[Title/Abstract] OR "embolotherap*"[Title/Abstract] OR "therapeutic embolization*"[Title/Abstract]) | **64** |
| 2 | "ablation technique*"[MeSH Terms] OR ("embolization*"[All Fields] AND "therapeutics"[MeSH Terms]) OR "Ablation"[Title/Abstract] OR ("technique*"[All Fields] AND "Ablation"[Title/Abstract]) OR "Embolization"[Title/Abstract] OR "embolotherap*"[Title/Abstract] OR "therapeutic embolization*"[Title/Abstract] | 257545 |
| 1 | "Hyperaldosteronism"[MeSH Terms] OR "Aldosteronism"[Title/Abstract] OR "primary hyperaldosteronism"[Title/Abstract] OR "hyperaldosteronism primary"[Title/Abstract] | 10954 |

Embase search strategy

| Search number | Search Details | Results |
| --- | --- | --- |
| #14 | #12 AND #13 | **153** |
| #13 | #5 OR #6 OR #7 OR #8 OR #9 OR #10 OR #11 | 300937 |
| #12 | #1 OR #2 OR #3 OR #4 | 17117 |
| #11 | 'therapeutic embolization*':ti,ab | 0 |
| #10 | 'embolotherap*':ti,ab | 1149 |
| #9 | 'embolization':ti,ab | 73315 |
| #8 | 'artificial embolization'/exp | 109353 |
| #7 | 'technique*, ablation':ti,ab | 0 |
| #6 | 'ablation':ti,ab | 165131 |
| #5 | 'ablation therapy'/exp | 61170 |
| #4 | 'hyperaldosteronism,primary':ti,ab | 15 |
| #3 | 'primary hyperaldosteronism':ti,ab | 2557 |
| #2 | 'aldosteronism':ti,ab | 6873 |
| #1 | 'hyperaldosteronism'/exp | 16190 |

Cochrane library search strategy

| Search number | Search Details | Results |
| --- | --- | --- |
| #1 | MeSH descriptor: [Hyperaldosteronism] explode all trees | 79 |
| #2 | (Aldosteronism):ti,ab,kw (Word variations have been searched) | 5411 |
| #3 | (Primary Hyperaldosteronism):ti,ab,kw (Word variations have been searched) | 127 |
| #4 | (Hyperaldosteronism, Primary):ti,ab,kw (Word variations have been searched) | 127 |
| #5 | #1 or #2 or #3 or #4 | 5441 |
| #6 | MeSH descriptor: [Ablation Techniques] explode all trees | 6298 |
| #7 | (Ablation):ti,ab,kw (Word variations have been searched) | 11078 |
| #8 | (Technique*, Ablation):ti,ab,kw (Word variations have been searched) | 0 |
| #9 | MeSH descriptor: [Embolization, Therapeutic] explode all trees | 866 |
| #10 | (Embolization):ti,ab,kw (Word variations have been searched) | 11578 |
| #11 | (Embolotherap*):ti,ab,kw (Word variations have been searched) | 37 |
| #12 | (Therapeutic Embolization*):ti,ab,kw (Word variations have been searched) | 698 |
| #13 | #6 or #7 or #8 or #9 or #10 or #11 or #12 | 26410 |
| #14 | #5 and #13 | **53** |
