# Supplementary figures and images for: The outcomes of transcatheter adrenal ablation in patients with primary aldosteronism: a systematic review and meta-analysis
Source: BMC Endocr Disord. 2023 May 8;23:103. doi: 10.1186/s12902-023-01356-9 (PMC10165838; doi:10.1186/s12902-023-01356-9)

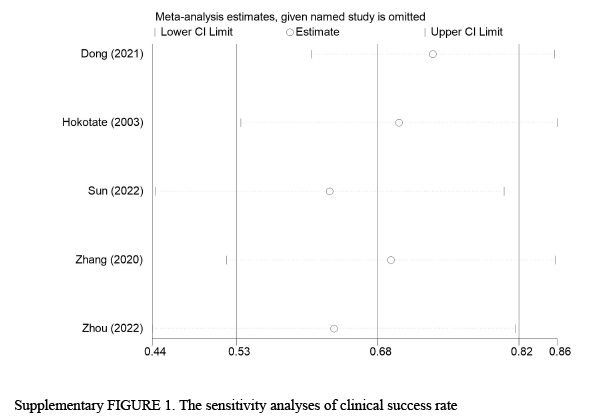

Supplement: Supplementary file 2 — Additional file 2: Figure S1. [file 12902_2023_1356_MOESM2_ESM.tif]

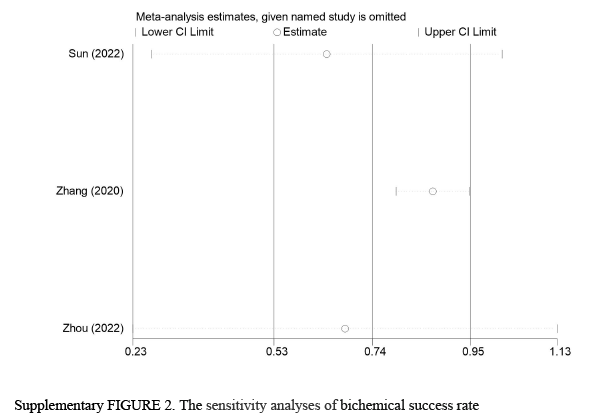

Supplement: Supplementary file 3 — Additional file 3: Figure S2. [file 12902_2023_1356_MOESM3_ESM.tif]
